# Piloting the Adult Protective Services’ Identification, Services, and Outcomes Matrix: Focus Group Findings

**DOI:** 10.1093/geroni/igaa057.153

**Published:** 2020-12-16

**Authors:** Jarmin Yeh, Pi-Ju Liu, Jacques Perkins, Andrew Butler, Sara Stratton, Kendon Conrad, Karen Conrad, Madelyn Iris

**Affiliations:** 1 University of California, San Francisco, San Francisco, California, United States; 2 Purdue University, West Lafayette, Indiana, United States; 3 San Francisco Adult Protective Services, San Francisco, California, United States; 4 University of Illinois at Chicago, Chicago, Illinois, United States; 5 University of Illinos at Chicago, Chicago, Illinois, United States; 6 Northwestern University, Chicago, Illinois, United States

## Abstract

The historical lack of outcomes-related data in Adult Protective Services (APS) has affected funding coming to the program. Without data quantifying the effectiveness of APS services, policymakers have been unable to justify budget increases to improve wages for workers or professionalize the field. For the first time in APS history, the U.S. Administration for Community Living sponsored a pilot project to implement a novel evidence-based assessment tool into APS electronic systems, called the Identification, Services, and Outcomes (ISO) Matrix. The goal was to improve APS’ ability to reduce harm of abuse and neglect and maintain client’s independence to live in the community. APS workers in San Francisco and Napa Counties were trained and phased into using the ISO Matrix over a six-month implementation period. This poster presents findings from six focus groups conducted between February 13 and March 28, 2019, with 34 San Francisco and Napa County APS workers and supervisors. Utilizing semi-structured, in-depth interviewing techniques, APS workers and supervisors expressed their views and experiences. Facilitators and barriers of implementing the ISO Matrix were assessed and opportunities for improvements were identified. Findings revealed a tension between their hopefulness that the ISO Matrix could modernize APS data-tracking and yield evidence of improved client outcome to bolster the field, and their frustrations about new burdens the ISO Matrix placed on their labor and workflow. Understanding frontline perspectives of APS workers and supervisors has practical and policy implications for adopting the ISO Matrix in other counties and states across the U.S.

